# Clinical and NGS predictors of response to regorafenib in recurrent glioblastoma

**DOI:** 10.1038/s41598-022-20417-y

**Published:** 2022-09-28

**Authors:** Silvia Chiesa, Antonella Mangraviti, Maurizio Martini, Tonia Cenci, Ciro Mazzarella, Simona Gaudino, Serena Bracci, Antonella Martino, Giuseppe M. Della Pepa, Martina Offi, Marco Gessi, Rosellina Russo, Matia Martucci, Francesco Beghella Bartoli, Luigi M. Larocca, Liverana Lauretti, Alessandro Olivi, Roberto Pallini, Mario Balducci, Quintino Giorgio D’Alessandris

**Affiliations:** 1 Department of Radiation Oncology, Fondazione Policlinico Universitario Agostino Gemelli IRCCS, Università Cattolica del S. Cuore, Largo A. Gemelli, 8, 00168 Rome, Italy; 2 Department of Neurosurgery, Fondazione Policlinico Universitario Agostino Gemelli IRCCS, Università Cattolica del S. Cuore, Largo A. Gemelli, 8, 00168 Rome, Italy; 3 Depatrment of Pathology, Fondazione Policlinico Universitario Agostino Gemelli IRCCS, Università Cattolica del S. Cuore, Largo A. Gemelli, 8, 00168 Rome, Italy; 4 Department of Radiology, Fondazione Policlinico Universitario Agostino Gemelli IRCCS, Università Cattolica del S. Cuore, Largo A. Gemelli, 8, 00168 Rome, Italy

**Keywords:** Genomic analysis, High-throughput screening, Cancer genomics, Cancer therapy, CNS cancer, Cancer genomics, Clinical genetics, Gene regulation, Genomics, Sequencing, Gliogenesis, Biological techniques, Biotechnology, Cancer, Genetics, Molecular biology, Neuroscience, Biomarkers, Predictive markers, Prognostic markers, Oncology, Cancer, Surgical oncology

## Abstract

Predictive factors for response to regorafenib in recurrent glioblastoma, IDH-*wildtype,* are scarcely recognized. The objective of this study was to identify molecular predictive factors for response to regorafenib using a clinically available platform. We analyzed a prospective cohort of 30 patients harboring recurrent glioblastoma, *IDH-*wildtype, and treated with regorafenib. Next-generation sequencing (NGS) analysis was performed on DNA extracted from paraffin-embedded tissues using a clinically available platform. Moreover, MGMT methylation and EGFRvIII expression analyses were performed. Six-month progression-free survival (PFS) was 30% and median overall survival (OS) was 7.5 months, in line with literature data. NGS analysis revealed a mutation in the EGFR pathway in 18% of cases and a mutation in the mitogen-activated protein-kinase (MAPK) pathway in 18% of cases. In the remaining cases, no mutations were detected. Patients carrying MAPK pathway mutation had a poor response to regorafenib treatment, with a significantly shorter PFS and a nonsignificantly shorter OS compared to EGFR-mutated patients (for PFS, 2.5 *vs* 4.5 months, *p* = 0.0061; for OS, 7 *vs* 9 months, *p* = 0.1076). Multivariate analysis confirmed that MAPK pathway mutations independently predicted a shorter PFS after regorafenib treatment (*p* = 0.0188). The negative prognostic role of MAPK pathway alteration was reinforced when we combined EGFR-mutated with EGFRvIII-positive cases. Recurrent glioblastoma tumors with an alteration in MAPK pathway could belong to the mesenchymal subtype and respond poorly to regorafenib treatment, while EGFR-altered cases have a better response to regorafenib. We thus provide a molecular selection criterion easy to implement in the clinical practice.

## Introduction

Treatment of recurrent glioblastoma (GBM), IDH-wildtype, after surgery and radiotherapy with concomitant and adjuvant temozolomide remains a challenge. Failure of many trials with targeted drugs, including bevacizumab, led to the adoption of lomustine as standard second-line treatment in Europe^1^. The introduction of regorafenib changed this scenario. Regorafenib is an orally available multikinase inhibitor, whose main targets are kinases involved in angiogenesis (VEGFR1–3 and TIE2), oncogenesis (KIT, RET, RAF1, and BRAF), tumor microenvironment (PDGFR and FGFR), and tumor immunity (colony stimulating factor 1 receptor)^2,3^. In the REGOMA trial^4^, patients with recurrent GBM were randomized to receive regorafenib or lomustine. The regorafenib arm showed a significantly longer overall survival (OS), both in terms of median OS (7.4 *vs* 5.6 months, respectively; *p* = 0.0009) and 12-month OS (38.9% *vs* 15%, respectively). Safety was deemed acceptable. On these bases, regorafenib has become the standard second-line treatment for GBM, *IDH-*wildtype in our country. This notwithstanding, the setting of recurrence is particularly hard, and many issues remain unresolved. e.g., in the REGOMA trial, at 6 months from the start of regorafenib more than 80% of patients had experienced disease progression. It is therefore mandatory to identify those patients who are likely to respond poorly to regorafenib. Due to its novelty, available literature data are scarce^5,6^. The objective of this study was to analyze the molecular profile of a cohort of patients suffering from recurrent GBM, IDH-wildtype, treated with regorafenib, with the aim to identify factors that may predict response to this multikinase inhibitor.

## Methods

### Study outline

We prospectively enrolled patients harboring recurrent GBM, *IDH-*wildtype, after surgery and standard chemoradiotherapy according with the Stupp protocol^7^, and treated with regorafenib in the multidisciplinary Neuro-Oncologic Board at Fondazione Policlinico Universitario “A. Gemelli”, Rome, Italy. Indications and schedule of regorafenib treatment followed the executive decision of the Italian Agency for Medicines 143345/2019. Regorafenib was administered orally at a dose of 160 mg/day for the first 3 weeks of each 4-week cycle^4^. Neurosurgery for recurrent tumor before regorafenib treatment was allowed, when possible, to confirm the diagnosis of tumor recurrence. The study followed the principles set forth in the World Medical Association Declaration of Helsinki and was approved by the local Institutional Ethics Committee (Prot. ID 3458). Informed consent was obtained from all individual participants included in the study.

### Standard histopathology and molecular profiling of the tumor

Enrolled patients were diagnosed with GBM, *IDH-*wildtype, according to WHO classification of tumours of the Central Nervous System, revised 4th edition^8^. The presence of *IDH1* R132H mutation was assessed by immunohistochemistry using the monoclonal antibody clone H09 (Dianova, Hamburg, Germany). MGMT promoter methylation analysis was performed with methylation-specific PCR, and EGFRvIII expression was assessed by RT-PCR, as previously described^9^.

### Analysis of next-generation sequencing data for sequence variants and copy number changes

Next-generation sequencing (NGS) analysis was performed on DNA extracted from paraffin-embedded tissues with a percentage of tumor cells higher than 50%, using the QIAcube system (Qiagen, Hilden, Germany). The DNA was then amplified using Myriapod NGS Cancer panel DNA, REF NG033, Kit IVD (Diatech Pharmacogenetics, Jesi, Italy) on EasyPGX qPCR instrument 96 (Diatech Pharmacogenetics), and the sequencing was done using Seq100 (Illumina, San Diego, CA). The sensitivity of the procedure was around 5%. The analyzed genes were ALK, BRAF, EGFR, ERDD2, FGFR3, HRAS, IDH1, IDH2, KIT, KRAS, MET, NRAS, PDGFR, PIK3CA, RET, ROS1. Data analysis was performed using Myriapod NGS Data Analysis Software and Myriapod NGS Workstation (Diatech Pharmacogenetics).

### Statistical analysis

Response to treatment was evaluated using Response Assessment in Neuro-Oncology (RANO) criteria^10^. Progression-free survival (PFS) and OS were calculated from regorafenib start to, respectively, progression of disease and patient’s death. The primary endpoint of the study was the correlation between molecular profile and PFS after regorafenib treatment. Secondary endpoints were the correlation between molecular profile and OS and response to treatment. Comparison of categorical variables was conducted with the Chi-square statistics, using the Fisher Exact test when appropriate. Comparison of continuous variables between groups was conducted using the Mann–Whitney *U* test. For survival analysis, Kaplan–Meier curves were plotted and comparison between groups was performed using the log-rank test. In the whole series, multivariate analysis of prognostic factors for PFS and OS was performed by using the Cox model, adjusted for the following parameters, age (< 65 years or ≥ 65 years), extent of resection, MGMT promoter methylation, EGFRvIII status, NGS analysis. Another multivariate analysis of prognostic factors for PFS and OS in patients with MAPK or EGFR pathway alteration was performed by using the Cox model, adjusting for the following parameters: age (< 65 years or ≥ 65 years), extent of resection, MGMT promoter methylation, molecular status. Median follow-up was calculated using the reverse Kaplan–Meier method. A *p* value less than 0.05 was considered as statistically significant. Analyses were performed using StatView ver 5.0 (SAS Institute, Cary, NC).

### Ethics approval

The study followed the principles set forth in the World Medical Association Declaration of Helsinki and was approved by the Institutional Ethics Committee of Fondazione Policlinico Gemelli IRCCS (Prot. ID 3458). We confirm that informed consent was obtained from all from all subjects (and/or their legal guardian(s)) included in the study.

## Results

### Baseline patients characteristics

Thirty recurrent GBM patients after standard radiotherapy with concomitant and adjuvant chemotherapy according to the Stupp protocol were considered in this study. NGS analysis showed that one of the patients harbored IDH-mutant GBM that was not detected using immunohistochemistry. Therefore, this patient was excluded from the study. Then, the final group was composed of 29 patients harboring GBM, IDH-wildtype (Online Resource 1, Supplementary Table S1).

At enrollment, the mean age was 58.4 ± 10.8 years. All patients had a good performance status (i.e., Karnofsky performance status ≥ 70). Fourteen patients (48.3%) had undergone surgery for recurrent GBM. At last surgery, gross-total tumor resection had been performed in 22 out of 29 cases (75.9%), whereas subtotal resection or biopsy in 7/29 cases (24.1%). Among patient who underwent surgery for recurrence, gross-total tumor resection was performed in 10 out of 14 cases (71.4%), whereas subtotal resection in 4/14 cases (28.6%). MGMT promoter was methylated in 12 out of 29 cases (41.4%) and unmethylated in 17/29 (58.6%). RT-PCR for EGFRvIII mRNA was available for 28 patients and was positive in 14 cases.

### Treatment results

Median follow-up was 20 months (range, 1.5–20). Dose reduction was required in 8 patients; reasons were thrombocytopenia (2 cases), fatigue (2 cases), hand-foot syndrome (2 cases), diarrhoea (1 case), and hyperbilirubinemia (1 case). At last follow-up, 8 out of 29 patients (27.5%) were alive, two of them with ongoing regorafenib treatment while the other six patients experienced disease progression during regorafenib treatment; 21/29 patients (72.5%) were dead. The median PFS of the whole cohort was 3 months. Six-month PFS (PFS-6) with regorafenib treatment was reached in 8 out of 27 patients (29.6%) and was not reached in 19/27 patients (70.4%). Median OS was 7.5 months. The best response was assessed using RANO criteria^10^, in which partial response (PR) requires ≥ 50% reduction of contrast enhancement without increase in FLAIR alterations, progressive disease (PD) requires a ≥ 25% increase of contrast enhancement or increase in FLAIR alterations or any new contrast enhancing lesion, and stable disease (SD) does not qualify either for PR or for PD. Excluding the 2 cases with ongoing regorafenib treatment, the best response was partial response (PR) in 5 cases (18.5%), stable disease (SD) in 8 cases (29.6%), and progressive disease (PD) in 7 cases (25.9%). In 7 other cases (25.9%), PD was diagnosed on clinical basis only.

### Next-generation sequencing analysis

NGS analysis was successfully conducted in 28 out of 29 cases. In 1 case, extracted DNA was not sufficient. In 5 out of 28 cases (17.9%), mutation of EGFR and/or PIK3CA was found. These cases were grouped together, as cases harboring mutation in the EGFR pathway. RAS mutations were found in two cases (7.1%) and RET mutations were found in 3 cases (10.1%). These cases were grouped together as cases harboring mutation in the mitogen-activated protein kinase (MAPK) pathway. In the remaining 18 cases (64.3%), no mutations of the analyzed genes were found. Details on the specific mutations are given in Online Resource 1, Supplementary Table S2.

### Standard prognostic factors for progression-free and overall survival after regorafenib treatment

Age had no prognostic value, probably due to the careful patients’ selection (Online Resource 2, Supplementary Fig. S1). Gross-total resection (GTR), instead, was endowed with a positive prognostic value for PFS (median PFS 4 months in GTR cases *vs* 2.5 months in subtotal resection/biopsy cases, *p* = 0.0296, log-rank test) but not for OS (Online Resource 2, Supplementary Fig. S1). Patients whose tumors had a methylated MGMT promoter had a median PFS of 4 months, while in patients with unmethylated MGMT promoter median PFS was 3 months, however, this difference was not significant (*p* = 0.42, log-rank test) (Online Resource 2, Supplementary Fig. S1). MGMT promoter methylation did not impact on OS (median OS 7.5 months both in methylated and unmethylated cases, *p* = 0.13, log-rank test). EGFRvIII expression conferred a not significantly longer PFS (median PFS 4 months in EGFRvIII positive *vs* 2.5 months in EGFRvIII negative cases, *p* = 0.40, log-rank test) and a significantly longer OS after regorafenib treatment (median PFS 8.5 months in positive *vs* 4.5 months in negative cases, *p* = 0.0131, log-rank test) (Online Resource 2, Supplementary Fig. S1).

### Prognostic role of next-generation sequencing analysis after Regorafenib treatment

**Figure 1:**
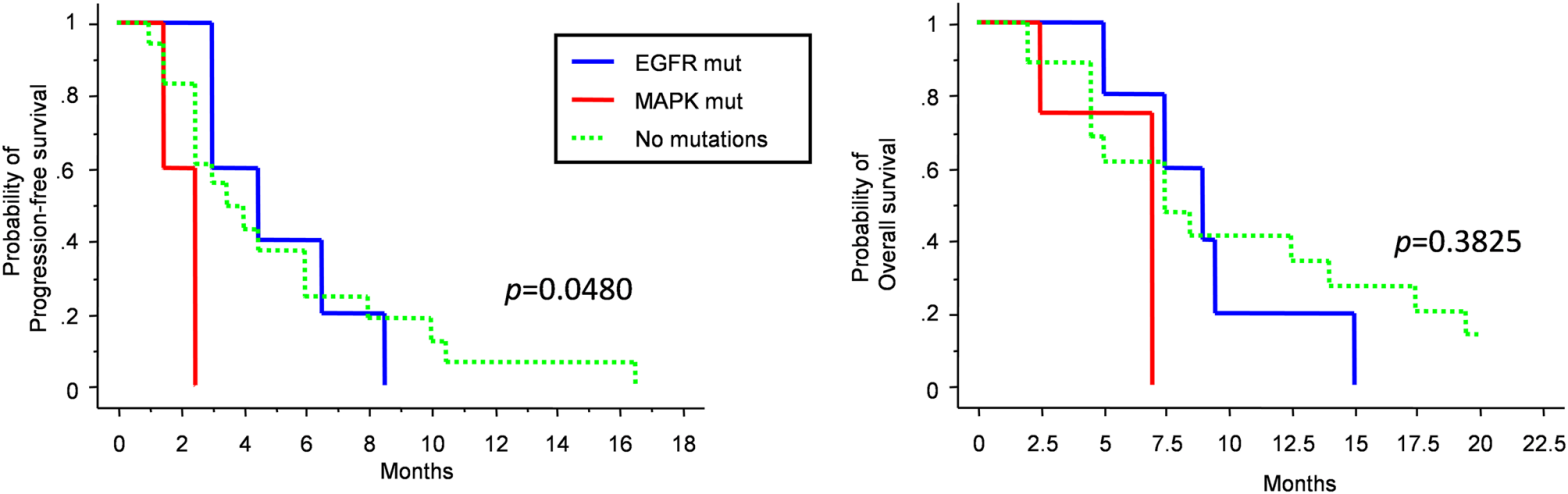
Kaplan–Meier survival curves for PFS (left) and OS (right) depending on pathway mutation at next-generation sequencing analysis.

Based on the analyzed genes, we categorized patients into three groups, those carrying a mutation in the EGFR pathway (*n* = 5), those carrying a mutation in the MAPK pathway (*n* = 5), and those with no detectable mutations (*n* = 18). Surprisingly, patients carrying a MAPK pathway mutation had a poor response to regorafenib treatment, with median PFS 2.5 months, which was significantly shorter compared to patients carrying EGFR pathway mutation (4.5 months) and wild-type cases (3.5 months) (*p* = 0.0480; *p* = 0.0061 for comparison between MAPK and with EGFR mutated patients; log-rank test) (Fig. 1). Overall survival analysis confirmed that patients harboring MAPK pathway mutation had shorter OS than those with EGFR pathway mutation, but the difference was not significant (median OS 7 *vs* 9 months, respectively; *p* = 0.1076; log-rank test).

Multivariate Cox analysis for PFS confirmed the independent negative predictive role of MAPK pathway alteration for response to regorafenib treatment (*p* = 0.0188; Table 1), while no independent prognostic factors for OS were identified (Online Resource 1, Supplementary Table S3).

**Table 1:** Multivariate analysis of factor affecting progression-free survival in the whole cohort.

Covariate	Hazard ratio	Confidence interval	*p-*value
MGMT	0.535	0.170–1.683	0.2849
Extent of resection	0.241	0.065–0.889	0.0326
EGFRvIII	2.2556	0.666–9.814	0.1715
Age	0.841	0.317–2.230	0.7274
MAPK pathway mutation	5.485	1.325–22.703	0.0188

### Prognostic role of combination of NGS-EGFRvIII analysis after regorafenib treatment

**Figure 2:**
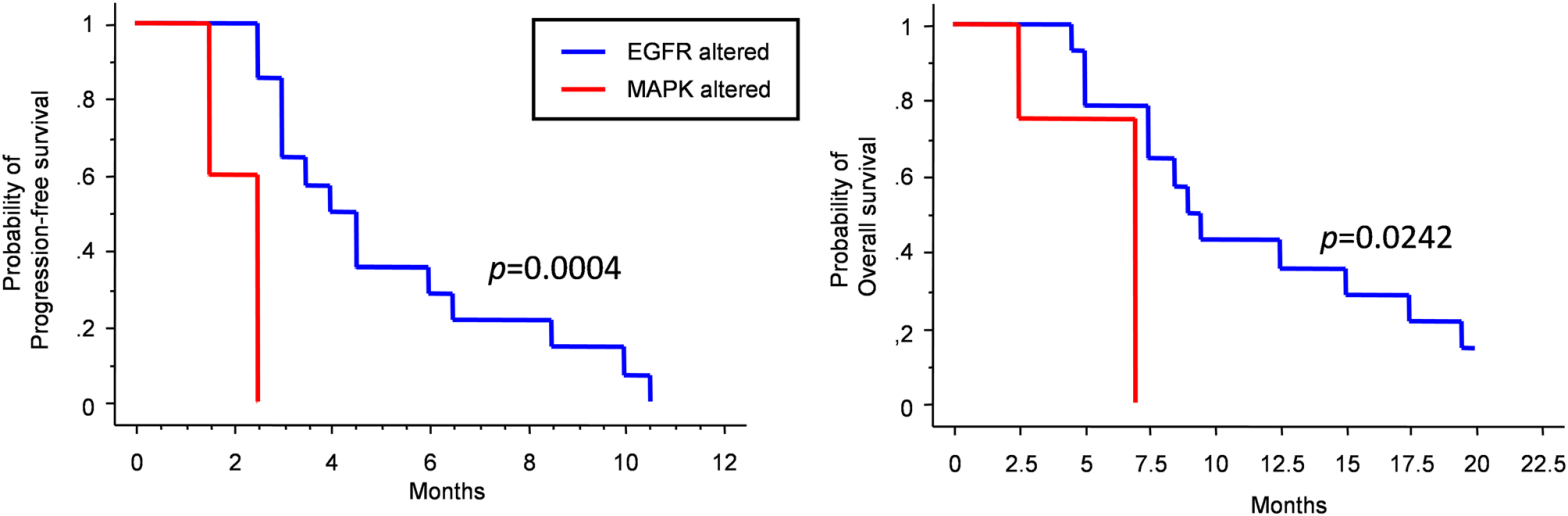
Kaplan–Meier survival curves for PFS (left) and OS (right) depending on pathway alteration at next-generation sequencing analysis combined with EGFRvIII status.

In 18 out of 28 cases (64%), NGS analysis did not identify any gene mutation; however, 9 of them (50%) expressed EGFRvIII as showed by RT-PCR analysis, portending an EGFR pathway activation. Therefore, we grouped these 9 patients together with the 5 cases harboring activating mutation of the EGFR pathway (*n* = 14 overall), and compared PFS and OS of this group with survival of patients harboring MAPK pathway mutation (n = 5). As shown in Fig. 2, patients with MAPK pathway alteration had significantly worse PFS and OS than those with an EGFR pathway alteration (median PFS 2.5 months *vs* 4 months, respectively; *p* = 0.0004; median OS 7 *vs* 9 months, respectively; *p* = 0.0242; log-rank test). Multivariate analysis confirmed the independent negative prognostic role of MAPK pathway activation compared to EGFR pathway activation, both for PFS (*p* = 0.0034; Table 2) and OS (*p* = 0.0426; Online Resource 1, Supplementary Table S4).

**Table 2:** Multivariate analysis of factors affecting progression-free survival in patients harboring EGFR or MAPK pathways alteration.

Covariate	Hazard ratio	Confidence interval	*p-*value
MGMT	1.076	0.312–3.710	0.9080
Age	1.081	0.348–3.357	0.8922
Extent of resection	0.320	0.075–1.369	0.1243
Pathway alteration	0.060	0.009–0.395	0.0034

## Discussion

Glioblastoma recurrence is one of the main challenges in neuro-oncology. Various salvage strategies have been applied with limited success. The randomized multicenter Phase II REGOMA trial showed that regorafenib significantly improved OS when compared to lomustine (7.4 *vs* 5.6 months)^4^. Considering the extreme heterogeneity of this tumor, we performed NGS analysis of a panel of genes in patients suffering from recurrent GBM, *IDH-*wildtype treated with regorafenib, to identify predictive factors for response to treatment. Few studies in the literature are available on this topic^5,6^. Remarkably, we observed a median PFS of 3 months and a median OS of 7.5 months, comparable to those reported in the REGOMA trial^4^.

The main result of our study is that patients harboring an alteration in the MAPK pathway, as assessed by NGS at DNA level, display poor response to regorafenib in terms of PFS, which was the primary endpoint of the study. Combining NGS analysis with RT-PCR for EGFRvIII, MAPK-altered patients had a significantly worse prognosis than EGFR-altered patients in terms of OS.

### Standard clinical and molecular prognostic factors of response to regorafenib

Noteworthy, all patients had KPS ≥ 70 at enrollment; it is thus not surprising that age was not a prognostic factor in this group, differently from unselected GBM cohorts. According with recent literature^11,12^, gross-total resection positively impacted on the clinical outcome. No patient undergoing subtotal resection or biopsy reached 6-month PFS. When considering separately patients who underwent surgery for recurrence, a trend to improved PFS with gross-total resection was confirmed at log-rank analysis (*p* = 0.0809).

We found higher PFS-6 rate in MGMT methylated than unmethylated patients, though PFS advantage at Kaplan–Meier analysis was not significant. This evidence has not an obvious mechanistic explanation. In the REGOMA trial, survival advantage with regorafenib was noticed both in unmethylated and methylated tumors with a greater amplitude in the latter^4^. Intriguingly, EGFRvIII mRNA expression conferred a nonsignificantly longer PFS and a significantly longer OS after regorafenib treatment. This evidence also is puzzling, since regorafenib is not an EGFR inhibitor. In a previous work from our group on a cohort of 73 primary GBM patients^9^, EGFRvIII-positive patients had median OS of 19 months, which was significantly longer than EGFRvIII negative ones. Thus, we could argue that the prognostic advantage of EGFRvIII-positive patients was independent from regorafenib treatment. In the present series, however, the cumulative OS, as calculated since GBM diagnosis, was not significantly different between EGFRvIII-positive and negative cases (22.5 *vs* 22 months, respectively; *p* = 0.7; log-rank test). It should be underlined that the work by Montano et al^9^ included a large cohort of unselected patients, whereas in the present study the patients were selected for their clinical conditions (KPS ≥ 70). Therefore, the EGFRvIII expression was a definite predictor of good response to regorafenib.

### Advanced molecular prognosticators of response to regorafenib

Regorafenib is a multikinase inhibitor, whose main targets are the VEGF receptors, the members of the ERK-MAPK pathways (RET, RAF-1, BRAF) and other kinases like KIT, PDGFR and FGFR. Due to its recent introduction into neuro-oncology practice, molecular factors for response are poorly known. The Padua group, which led the REGOMA trial, published two papers where, (i) the immunohistochemical expression of phosphorylated acetyl-CoA carboxylase(pACC)^6^, and (ii) the mini-signature including HIF1A and CDKN1A mRNAs, besides 3 miRNAs, showed positive predictive value for response to regorafenib^5^. Mechanistically, HIF1A, CDKN1A, and the 3 miRNAs are angiogenesis players: their positive predictive value for response to regorafenib is therefore explained by the anti-angiogenic activity of regorafenib. Antiangiogenic treatment would trigger the 5’adenosine monophosphate-activated protein kinase (AMPK) and prime metabolic rewiring, which restrains cell proliferation. Interrogating ACC phosphorylation in tumors can indicate whether the pathway is active. Therefore, pACC-positive tumors are thought to be more angiogenic compared with pACC-negative ones. However, this hypothesis was not supported by microvasculature density analysis, which suggested similar angiogenesis in pACC-positive and pACC-negative tumors. In the pAMPK-positive cohort of patients treated with regorafenib, there was improved OS, but this biomarker yielded negative results in the interaction test.

In our analysis, we identified mutations in 36% of GBM tumors, which involved the EGFR, PIK3CA, Ras and RET genes (Online Resource 3, Supplementary Discussion). EGFR/PI3KA mutations are one of the most frequent events in gliomagenesis^13^. Ras is a known player in the ERK-MAPK pathways^14^. RET is a receptor tyrosine kinase, whose dimerization and subsequent internalization activates a variety of downstream pathways, including the MAPK/ERK pathway^14^. Reportedly, the RET mutations that were found in this study activate specifically the MAPK pathway (Online Resource 3, Supplementary Discussion)^15^. Then, it is somewhat counter-intuitive that activating mutations of this pathway, which is a known target of regorafenib, are predictive of poor response. While the scarcity of available data hampers a definitive explanation, some speculations can be made. We might postulate that the mutations we found render the tyrosine kinases RET and RAF (the downstream effector of Ras) resistant to regorafenib inhibition^16,17^. Another intriguing explanation concerns the ERK-MAPK pathway, which is regarded as the core pathway of mesenchymal GBM^18^. It is not surprising that regorafenib, an anti-angiogenic drug, has a scarce activity on mesenchymal GBM. Bevacizumab, another anti-angiogenic drug, is reportedly more effective on proneural GBMs^19^. The mesenchymal subtype of GBM displays higher metabolic flexibility with both glycolytic and oxidative metabolisms. In contrast to proneural ones, the mesenchymal GBMs easily bypass targeted metabolic inhibition through a wide range of metabolic adaptation. To overcome their metabolic flexibility, an efficient metabolic targeting of mesenchymal GBM will require the blockade of several metabolic pathways. For example, in preclinical cancer models, dual inhibition of both glycolysis and oxidative phosphorylation has been shown to effectively reduce tumor growth^20^. To conclude, the NGS panel used in this analysis could identify a subgroup of tumors with activated MAPK pathway, putatively mesenchymal recurrent GBMs, which responded poorly to regorafenib.

### Strength of the present study

We present a series of patients homogeneously treated at a nationwide neuro-oncologic referral center, where clinical decisions and follow-up are regularly made by the multidisciplinary neuro-oncologic team. The prospective enrollment of patient assures the high quality of data. Another strong point of this work is linked to the NGS platform (Illumina ISeq™ 100). This is a rapid system, which does not require demanding hardware, and thanks to CE/IVD validation can be used for diagnostic purpose. The system allows the sequencing of small groups of patients per run (8–10 patients) and has reduced costs (about 150 euro per patient). Therefore, it allows time-sensitive genomic analysis of patients candidate to chemotherapy.

### Limitations of the study

The monocentric nature of the study can be regarded as a limitation, though, as stated before, it assures the quality of clinical and molecular data. The sample size is relatively small, and this can impact on statistical significance. In this study, we used PFS as primary endpoint, while most studies on recurrent GBM use as endpoint the OS, in order to overcome uncertainties in assessing progression^4,21^. However, we believe that PFS is an adequate endpoint for the assessment of predictive factors for response to regorafenib, as it is not influenced by the responses to first-line treatment or to third-line therapies.

## Conclusions

Our data suggest that NGS analysis using a simple and cheap platform, combined with standard molecular profiling, might be used in patients candidate to second-line chemotherapy in order to select patients who can benefit from regorafenib treatment. Such approach could be useful in our clinical practice to customize the second-line strategy, taking into account patient’s comorbidities and the possible adverse events related to different treatments. Although far from being conclusive, these data support the development of further studies.

## Supplementary Information

Supplementary Information 1.

Supplementary Information 2.

Supplementary Information 3.

## Data Availability

The datasets analysed during the current study are available in the clinVAR repository (www.ncbi.nlm.nih.gov/clinvar/). The 3 variants not available in clinVAR repository have already been described (See Online Resource 1, Supplementary Table S2).

## Abbreviations

EGFR: Epidermal growth factor receptor
EGFRvIII: Epidermal growth factor receptor variant III
GBM: Glioblastoma
GTR: Gross-total resection
MAPK: Mitogen-activated protein-kinase
NGS: Next-generation sequencing
OS: Overall survival
PFS: Progression-free survival
PFS-6: Six-month progression-free survival
PD: Progressive disease
PR: Partial response
RANO: Response Assessment in Neuro-Oncology
SD: Stable disease
