# Supplementary material for: Clinical and NGS predictors of response to regorafenib in recurrent glioblastoma
Source: Sci Rep. 2022 Sep 28;12:16265. doi: 10.1038/s41598-022-20417-y (PMC9519741; doi:10.1038/s41598-022-20417-y)
Supplement: Supplementary file 1 — Supplementary Information 1. [file 41598_2022_20417_MOESM1_ESM.docx]

**Supplementary Table S1. Baseline characteristics of study patients**

| **Parameter** | **Value** |
| --- | --- |
| Age (mean ± SD) | 58.4 ± 10.8 years |
| Maximum enhancing tumor diameter (mean ± SD) | 3.6 ± 1.3 cm |
| KPS, median (range) | 90 (70-90) |
| Surgery at tumor recurrence  Yes  No | 14 (48.3%)  15 (51.7%) |
| Extent of resection at last surgery  GTR No GTR | 22 (75.9%)  7 (24.1%) |
| Extent of resection at surgery for recurrence  GTR No GTR | 10 (71.4%)  4 (28.6%) |
| MGMT promoter  Methylated  Unmethylated | 12 (41.4%)  17 (58.6%) |
| EGFRvIII  positive  negative | 14 (50%)  14 (50%) |

**Supplementary Table S2. Details of gene mutations identified at NGS analysis**

| **Gene** | **Mutation** | ***n* cases** | **Reference** |
| --- | --- | --- | --- |
| EGFR | Ex7: c.787A>C, p.Thr263Pro | 1 | ClinVAR |
| EGFR | EGFR ex 15: c.1784G>A p.Cys595Tyr | 1 | See Ref. S2 |
| EGFR | Ex15: c.1793G>T p.Gly598Val | 1 | ClinVAR |
|  | Ex19: c.2235_2249del p.Glu746_Ala750del |  |  |
| EGFR | EGFRex7 c.866C>T p.Ala289Val | 1 | ClinVAR |
| PIK3CA | Ex10:c.1652G>A p.Glu542Lys |  |  |
| PIK3CA | Ex21: c.3010A>G p.Met1004Val | 1 | See Ref. S5 |
| NRAS | EX2: c.35G>A, p.Gly12Asp | 1 | ClinVAR |
| HRAS | Ex3: c.187G>A, p.Glu63Lys | 1 | ClinVAR |
| RET | Ex10:c.1763G>A p.Gly588Asp | 1 | See Ref. S17 |
| RET | Ex11: c.2071G>A p.Gly691Ser | 2 | ClinVAR |

ClinVAR, [www.ncbi.nlm.nih.gov/clinvar/](http://www.ncbi.nlm.nih.gov/clinvar/) References refer to Supplementary References in the Supplementary Discussion.

**Supplementary Table S3. Multivariate analysis of factor affecting overall survival in the whole cohort**

| **Covariate** | **Hazard ratio** | **Confidence interval** | ***p-*value** |
| --- | --- | --- | --- |
| MGMT | 0.937 | 0.253-3.472 | 0.9224 |
| Extent of resection | 1.143 | 0.264-4.955 | 0.8582 |
| EGFRvIII | 3.695 | 0.651-20.963 | 0.1400 |
| Age | 1.097 | 0.357-3.373 | 0.8713 |
| MAPK pathway mutation | 2.844 | 0.471-17.159 | 0.4713 |

**Supplementary Table S4. Multivariate analysis of factors affecting overall survival in patients harboring EGFR or MAPK pathways alteration**

| **Covariate** | **Hazard ratio** | **Confidence interval** | ***p-*value** |
| --- | --- | --- | --- |
| MGMT | 1.754 | 0.421-7.304 | 0.4401 |
| Age | 1.527 | 0.428-5.454 | 0.5144 |
| Extent of resection | 1.364 | 0.305-6.113 | 0.6847 |
| Pathway alteration | 0.124 | 0.017-0.933 | 0.0426 |
