# Supplementary figures and images for: Clinical and NGS predictors of response to regorafenib in recurrent glioblastoma
Source: Sci Rep. 2022 Sep 28;12:16265. doi: 10.1038/s41598-022-20417-y (PMC9519741; doi:10.1038/s41598-022-20417-y)

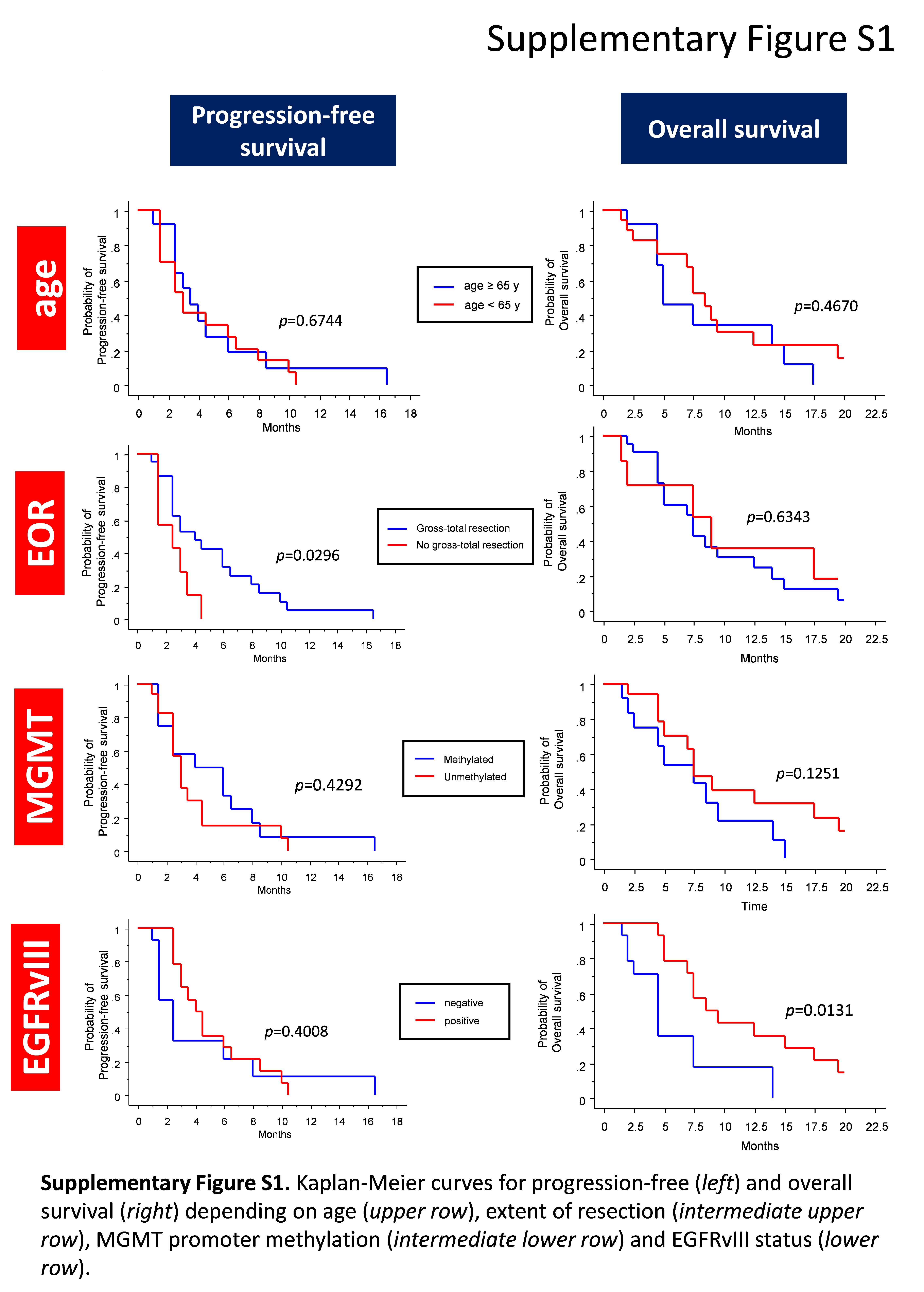

Supplement: Supplementary file 2 — Supplementary Information 2. [file 41598_2022_20417_MOESM2_ESM.tif]
