# Supplementary material for: Clinical and NGS predictors of response to regorafenib in recurrent glioblastoma
Source: Sci Rep. 2022 Sep 28;12:16265. doi: 10.1038/s41598-022-20417-y (PMC9519741; doi:10.1038/s41598-022-20417-y)
Supplement: Supplementary file 3 — Supplementary Information 3. [file 41598_2022_20417_MOESM3_ESM.pdf]

Supplementary Discussion

EGFR mutations are one of the most frequent events in gliomagenesis [S1,S2]. Phosphatidylinositol-4,5-bisphosphate 3-kinase, catalytic subunit alpha (PIK3CA) is a key player in the receptor tyrosine kinase pathway and, accordingly, has a key role in GBM tumorigenesis across the various subtypes. It is also one of the most frequently mutated genes in GBM [S1] with the highest percentages in proneural ones [S3, S4]. M1004V mutation lies within the PI3K/PI4K domain of the Pik3ca protein [S5] and has been described in several tumors, including endometrial [S6] and mammary angiosarcomas [S7]. E542K mutation has been largely described in a variety of cancers including GBM [S8].

The Ras family of GTPases encompases several members, including HRAS and NRAS, which are all players in the ERK-MAP kinase pathway [12]. The E63K pathogenic variant of the HRAS gene is reportedly associated with congenital myopathy [S9-S11] and has been described in medullary thyroid carcinoma [S12]. NRAS G12D mutation is a well-known oncogenic mutation in the MAP Kinase pathway and it has been detected in a variety of cancers including acute myeloid leukemia and colorectal adenocarcinoma [S8].

RET is a receptor tyrosine kinase whose dimerization and subsequent internalization activates a variety of downstream pathways, including the MAP kinase/ERK or, alternatively, the PI3 kinase/Akt [12]. RET G691S is a well-known mutation associated with multiple endocrine neoplasia syndrome and thyroid medullary carcinoma[S13]; it has also been reported in other cancers including pheocromocytoma [S14], lung adenocarcinoma [S15] and meningioma [S16]. Notably, in pancreatic cancer, RET G691S mutation triggers selectively the MAP Kinase/ERK pathway [13]. RET G588D is a less known mutation reported only in a study in lung adenocarcinoma [S17]. Aminoacid in position 588 belongs to a cysteine-rich domain important for receptor dimerization and it is a site of N-myristoilation[S18], thus influencing receptor internalization. In summary, literature data support the notion that the mutations of Ras and RET which have been detected in our patients activate the MAPK pathway.

Supplementary References

S1. Brennan CW, Verhaak RG, McKenna A, Campos B, Noushmehr H, Salama SR, et al (2013) The somatic genomic landscape of glioblastoma. Cell 155: 462-477. https://doi.org/710.1016/j.cell.2013.09.034
S2. Felsberg J, Hentschel B, Kaulich K, Gramatzki D, Zacher A, Malzkorn B, Kamp M, Sabel M, Simon M, Westphal M, Schackert G, Tonn JC, Pietsch T, von Deimling A, Loeffler M, Reifenberger G, Weller M; German Glioma Network (2017) Epidermal Growth Factor Receptor Variant III (EGFRvIII) Positivity in EGFR-Amplified Glioblastomas: Prognostic Role and Comparison between Primary and Recurrent Tumors. Clin Cancer Res 23: 6846-6855. https://doi.org/10.1158/1078-0432.CCR-17-0890.
S3. Verhaak RG, Hoadley KA, Purdom E, Wang V, Qi Y, Wilkerson MD, et al (2010) Integrated genomic analysis identifies clinically relevant subtypes of glioblastoma characterized by abnormalities in PDGFRA, IDH1, EGFR, and NF1. Cancer Cell 17: 98-110. https://doi.org/10.1016/j.ccr.2009.12.020
S4: Wang Q, Hu B, Hu X, Kim H, Squatrito M, Scarpace L, et al (2017) Tumor Evolution of Glioma-Intrinsic Gene Expression Subtypes Associates with Immunological Changes in the Microenvironment. Cancer Cell 32: 42-56.e6. https://doi.org/10.1016/j.ccell.2017.06.003
S5. UniProt.org. Accessed February 28, 2022.
S6. Torricelli F, Nicoli D, Bellazzi R, Ciarrocchi A, Farnetti E, Mastrofilippo V, Zamponi R, La Sala GB, Casali B, Mandato VD (2018) Computational development of a molecular-based approach to improve risk stratification of endometrial cancer patients. Oncotarget 9: 25517-25528.
https://doi.org/10.18632/oncotarget.25354

S7. Beca F, Krings G, Chen YY, Hosfield EM, Vohra P, Sibley RK, Troxell ML, West RB, Allison KH, Bean GR (2020) Primary mammary angiosarcomas harbor frequent mutations in KDR and PIK3CA and show evidence of distinct pathogenesis. Mod Pathol 33: 1518-1526. https://doi.org/10.1038/s41379-020-0511-6
S8. AACR Project GENIE Consortium (2017) AACR Project GENIE: Powering Precision Medicine through an International Consortium. Cancer Discov 7: 818-831. https://doi.org/10.1158/2159-8290.CD-17-0151
S9. van der Burgt I, Kupsky W, Stassou S, Nadroo A, Barroso C, Diem A, Kratz CP, Dvorsky R, Ahmadian MR, Zenker M (2007) Myopathy caused by HRAS germline mutations: implications for disturbed myogenic differentiation in the presence of constitutive HRas activation. J Med Genet 44: 459-462. https://doi.org/10.1136/jmg.2007.049270
S10. Bolocan A, Quijano-Roy S, Seferian AM, Baumann C, Allamand V, Richard P, Estournet B, Carlier R, Cavé H, Gartioux C, Blin N, Le Moing AG, Gidaro T, Germain DP, Fardeau M, Voit T, Servais L, Romero NB (2014) Congenital muscular dystrophy phenotype with neuromuscular spindles excess in a 5-year-old girl caused by HRAS mutation. Neuromuscul Disord 24: 993-998. https://doi.org/10.1016/j.nmd.2014.06.437
S11. Henry JM, Chahin N, Shiloh-Malawsky Y, Fan Z, Selcen D (2015) A rare case of congenital myopathy with excess muscle spindles: expanding the clinical spectrum of HRAS-associated neuromuscular disease. J Neurol 262: 1587-1589. https://doi.org/10.1007/s00415-015-7775-7
S12. Boichard A, Croux L, Al Ghuzlan A, Broutin S, Dupuy C, Leboulleux S, Schlumberger M, Bidart JM, Lacroix L (2012) Somatic RAS mutations occur in a large proportion of sporadic RET-negative medullary thyroid carcinomas and extend to a previously unidentified exon. J Clin Endocrinol Metab 97:E2031-E2035. https://doi.org/10.1210/jc.2012-2092. Epub 2012 Aug 3. PMID: 22865907; PMCID: PMC3462939.
S13. Colombo C, Minna E, Rizzetti MG, Romeo P, Lecis D, Persani L, Mondellini P, Pierotti MA, Greco A, Fugazzola L, Borrello MG (2015) The modifier role of RET-G691S polymorphism in hereditary medullary thyroid carcinoma: functional characterization and expression/penetrance studies. Orphanet J Rare Dis 10:25. https://doi.org/10.1186/s13023-015-0231-z
S14. Crona J, Verdugo AD, Granberg D, Welin S, Stålberg P, Hellman P, Björklund P (2013) Next-generation sequencing in the clinical genetic screening of patients with pheochromocytoma and paraganglioma. Endocr Connect 2: 104-111. https://doi.org/10.1530/EC-13-0009
S15. Osoegawa A, Hashimoto T, Takumi Y, Abe M, Yamada T, Kobayashi R, Miyawaki M, Takeuchi H, Okamoto T, Sugio K (2018) Acquired resistance to an epidermal growth factor receptor-tyrosine kinase inhibitor (EGFR-TKI) in an uncommon G719S EGFR mutation. Invest New Drugs 36: 999-1005. https://doi.org/10.1007/s10637-018-0592-y
S16. Jungwirth G, Warta R, Beynon C, Sahm F, von Deimling A, Unterberg A, Herold-Mende C, Jungk C (2019) Intraventricular meningiomas frequently harbor NF2 mutations but lack common genetic alterations in TRAF7, AKT1, SMO, KLF4, PIK3CA, and TERT. Acta Neuropathol Commun 7: 140. https://doi.org/10.1186/s40478-019-0793-4
S17. Zehir A, Benayed R, Shah RH, Syed A, Middha S, Kim HR, et al (2017) Mutational landscape of metastatic cancer revealed from prospective clinical sequencing of 10,000 patients. Nat Med 23: 703-713. https://doi.org/10.1038/nm.4333
S18. Atlas of Genetics and Cytogenetics in Oncology and Haematology. URL http://AtlasGeneticsOncology.org. Accessed February 28, 2022.
